# Comparative study of cytotoxicity of detonation nanodiamond particles with an osteosarcoma cell line and primary mesenchymal stem cells

**DOI:** 10.1080/13102818.2014.947704

**Published:** 2014-10-17

**Authors:** Milena Keremidarska, Aneliya Ganeva, Dimitar Mitev, Todor Hikov, Radina Presker, Lilyana Pramatarova, Natalia Krasteva

**Affiliations:** ^a^ Institute of Biophysics and Biomedical Engineering, Bulgarian Academy of Sciences, Sofia, Bulgaria; ^b^ School of Chemistry, Australian Centre for Research on Separation Science (ACROSS), University of Tasmania, Australia; ^c^ Georgi Nadjakov Institute of Solid State Physics, Bulgarian Academy of Sciences, Sofia, Bulgaria; ^d^ University of Ljubljana, Ljubljana, Slovenia

**Keywords:** nanodiamonds, cell proliferation, cell morphology, osteoblasts

## Abstract

Recently, nanodiamonds (NDs) have attracted great interest due to their unique physical and chemical properties that could be used in various biological applications. However, depending on the origin, NDs often contain different impurities which may affect cellular functions and viability. Therefore, before their biomedical application, the cytotoxicity of newly produced NDs should be assessed.

In the present study, we have evaluated cytotoxicity of four types of ND particles with two cell models: a human osteosarcoma cell line, MG-63, and primary rat mesenchymal stem cells (rMSCs). Detonation-generated nanodiamond (DND) particles were purified with different acid oxidizers and impurities’ content was determined by elemental analysis. The particles size distribution was measured revealing that the DND particles have an average size in the range of 51–233 nm. Cytotoxicity was assessed by optical microscopy and proliferation assay after 72 hours exposure of the cells to nanoparticles. We observed cell-specific and material-specific toxicity for all tested particles. Primary stem cells demonstrated higher sensitivity to DND particles than osteosarcoma cells. The most toxic were the DND particles with the smallest grain size and slight content of non-diamond carbon, while DNDs with higher grain size and free from impurities had no significant influence on cell proliferation and morphology. In addition, the smaller DND particles were found to form large aggregates mainly during incubation with rMSCs. These results demonstrate the role of the purification method on the properties of DND particles and their cytotoxicity as well as the importance of cell types used for evaluation of the nanomaterials.

## Abbreviations

NDs: Nanodiamonds
rMSCs: Rat mesenchymal stem cells
DND: Detonation-generatednanodiamond
HPHT: High-pressure high-temperature
CVD: Chemical vapour deposition
ICP-MS: Inductively coupled plasma mass spectrometer
ATCC: American type culture collection
DMEM: Dulbecco's modified Eagle's medium
EDTA: Ethylenediaminetetraacetic acid
FBS: Fetal bovine serum
CCK: Cell Counting Kit
WST: Water-soluble tetrazolium salt
PBS: Phosphate-buffered saline

## Introduction

Nanodiamonds (NDs) are small carbon particles with typical size around 2–10 nm. In the last years, they have received great interest in biomedical applications due to the attractive physicochemical and biological properties.[1–7] They combine the superior mechanical characteristics of diamond, such as chemical stability and extremely high hardness, stiffness and strength as well as the advantages of nanomaterials such as small size, large surface area and high adsorption capacity.[4,6] In addition, NDs have been shown to exhibit lower cytotoxicity compared to other carbon-based nanomaterials.[8]

NDs have been first found in meteorites and interstellar dust.[9] Today, there are numerous methods for synthetic production of NDs such as the detonation technique, laser ablation, high-energy ball milling of high-pressure high-temperature (HPHT) diamond microcrystals, plasma-assisted chemical vapour deposition (CVD), autoclave synthesis from supercritical fluids, chlorination of carbides, ion irradiation of graphite, electron irradiation of carbon ‘onions’ and ultrasound cavitation.[10–12] Among all of them the detonation of carbon-containing explosives seems to be one of the most cost-effective methods allowing large-scale production of ND powder for research and industrial application. The resultant product – detonation soot – is a mixture of diamond particles (up to 75 wt%) with other carbon allotropes (25–85 wt%) and incombustible impurities (metals and oxides, 1–8 wt%) that has to be purified for the most applications.[13,14] For removal of non-diamond carbon, the following chemicals can be used: ozone-enriched air, or liquid oxidants such as HNO_3_, HClO_4_ or different acid mixture under pressure, while metal impurities can be removed by treatment with HCl.[6] Since the purification procedures used by different manufacturers can influence ND properties, the cytotoxicity testing of newly derived ND particles should be done before their biomedical application to ensure that they will not affect cell viability and growth. Despite the growing interest in nanoparticles and their effect on the body, standardized procedures for the evaluation of nanoparticle toxicity have not been outlined yet and a lot of problems still have to be surmounted. One of them concerns the choice of a cell model. Most of the toxicity screening studies published so far reported that different cell types exhibited different sensitivity to nanoparticles indicating that the choice of a cell model influences the findings.[15–17] Therefore, the cell type must be selected considering the introduction route and target organ of the nanoparticle. In this study, we have used osteoblast cells and bone-marrow-derived mesenchymal stem cells because the tested DND particles will be further used in our future work to develop composite materials for bone tissue engineering. On the other hand, we wanted to compare primary cells to a cell line with the same origin to understand which cells are more sensitive to DND particles in order to obtain more comprehensive view about the biological impact of diamond particles. This information also could be useful for the correct choice of a cell model and for an accurate identification of nanomaterial cytotoxicity.

## Materials and methods

### Nanodiamond particles production and characterization

**Figure 1.**
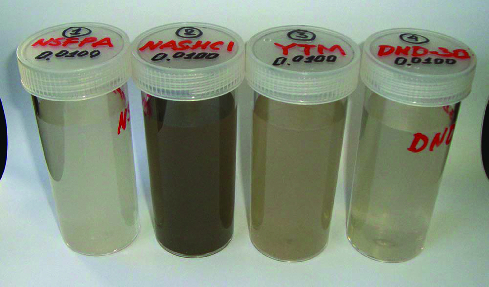
Water suspensions of the studied detonation nanodiamonds with concentration 0.1 mg/ml.

In this work we have studied four different types of detonation-generated nanodiamond particles denoted as NSFPA, NASHCl, YTM and DND-30 (Figure 1). Two of them (NSFPA and NASHCl) originated from the same primary detonation soot delivered from YTM ARGE A.S. (Istanbul, Turkey) which was further purified in University of Tasmania, Australia with different oxidizers: a mixture of HNO_3_, H_2_SO_4_, HClO_4_, HF with ratio 44%/44%/6%/6% for the sample 1 (NSFPA) and a mixture of HNO_3_, H_2_SO_4_, HCl with ratio 70%/20%/10% for the sample 2 (NASHCl) at temperatures up to 533 K and pressures up to 1.1 × 10^7^ Pa (in autoclave). Sample 3 (YTM) was delivered in already purified form from YTM ARGE A.S. (Istanbul, Turkey) where a mixture of H_2_SO_4_ and K_2_Cr_2_O_7_ has been used (according to the datasheet). The last sample 4 (DND-30) was purchased from Beijing Grish Hitech Co. (China) and there were no data about the used oxidizers.

DND suspensions were prepared as DND powders were suspended in distilled water at concentrations 0.1 mg/ml. Before cell experiments, DND suspensions were sterilized in an autoclave and were treated in an ultrasonic bath triply for 10 minutes in order to break the spontaneously formed aggregates into smaller particles.

Physicochemical characterization of DND particles was done by the measurement of particle size distribution using a Zetasizer Nano ZS particle analyser (ATA Scientific, Taren Point, NSW, AU) equipped with 632.8 nm red laser at 175° backscatter detection and by elemental analysis (ICP-MS measurements), using an ELEMENT 2 sector field inductively coupled plasma mass spectrometer (ICP-MS; Thermo Fisher, Bremen, Germany) with direct introduction of the produced diluted suspensions (0.1 mg g^−1^) to the instrument.

### Cells and cell culture conditions

Rat mesenchymal stem cells (rMSCs) were isolated and cultured according the centrifugation method of Dobson.[18] All protocols concerning the use of animals were approved by the Institutional ethical committee. In brief, bone marrow collected from the tibia and femur of two-month-old rats was centrifuged at 200 × *g* for 5 minutes. Supernatant, containing thrombocytes and erythrocytes, was discarded and the marrow pellet was resuspended in culture medium and plated in 25 cm^2^ tissue culture flasks at a density of 1 × 10^6^ cells/ml. After 72 hours non-adherent cells were removed and the medium was replaced every two to three days to allow further growth. A homogenous cell population was obtained after two weeks and after the first harvesting the cells were defined as passage zero (p0). The cells of the second and third passages were used in our experiments.

Human osteosarcoma cell line, MG-63, was obtained from American Type Culture Collection (ATCC, CRL-1427).

The cells were cultured in standard Dulbecco's modified Eagle's medium (DMEM), supplemented with 10% fetal bovine serum (FBS), 1 mM sodium pyruvate, 2 mM glutamine, 50 U/ml penicillin, 50 mg/ml streptomycin and 100 mM non-essential amino acids. All tissue culture reagents were purchased from Sigma-Aldrich Co. Cultured cells were propagated at 37 °C in a humidified 5% CO_2_ incubator to 80%–90% confluence, and after then were harvested with Trypsin-EDTA solution (0.25% Trypsin, 1 mM EDTA) and passage at a ratio 1:2 or seeded onto 24-well plates for the cytotoxicity test.

### Cell proliferation

To estimate the cytotoxic effect of DND particles on cells, we quantified the number of living cells using a Cell Counting Kit-8 (CCK-8, Sigma-Aldrich Co.). It is a non-radioactive, sensitive colorimetric assay, based on bioreduction of a highly water-soluble tetrazolium salt, WST-8 (2-(2-methoxy-4-nitrophenyl)-3-(4-nitrophenyl)-5-(2, 4-disulfophenyl)-2H-tetrazolium, monosodium salt) to a water-soluble formazan dye (yellow-coloured) in the presence of an electron carrier. The amount of the formazan dye generated by the activity of dehydrogenases in cells is directly proportional to the number of living cells.

For the assay the cells were seeded into 24-well plates at a density of 2 × 10^4^ cells per well and cultured for 24 hours in DMEM, supplemented with 10% FBS. On the next day, the culture medium was replaced with fresh one and 100 μl of each type of DND particles (dissolved in water) were added directly to the cell cultures. The cells were then incubated for three more days at 37 °C in a humidified 5% CO_2_ environment. At the 24th, 48th and 72th hours after addition of the particles, the cells were transferred to a new plate, washed once with PBS and proceeded for CCK-8 assay according to manufacturer's instructions. The formazan product was measured at 450 nm using a standard microplate reader (Infinite F200 Pro, TECAN, Austria GmbH). Cells not exposed to nanoparticles were used as a control.

### Cell morphology

To visualize the morphology of MG-63 and rMSCs, we have taken phase-contrast pictures at the 24th, 48th and 72th hours after addition of the DND suspensions, using an inverted microscope Axiovert 25 (Carl Zeiss, Germany). Phase-contrast microscopy is one of the easiest ways to monitor viable cells without staining and without loss of resolution. Therefore, we have used it here to detect any changes in cell morphology and cell viability after exposure of the cells to DND particles and thus to estimate cytotoxic effect of DNDs on cells.

### Statistical analysis

All experiments were done in triplicate and the results were represent as ± standard deviation wherever possible. The experimental data were analysed by ANOVA analysis. Statistical significance was accepted at a level of *p* < 0.05.

## Results and discussion

### Characterization of DND particles

**Figure 2.**
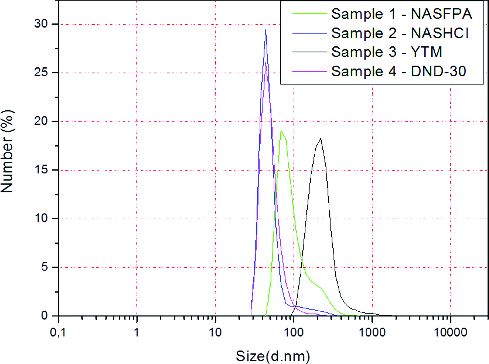
Size distribution of DND particles.

Physicochemical characterization of DND particles revealed a difference in particle size and content of metal impurities and non-diamond carbon, between all studied samples. Results from the measurements of particle size distribution are shown in Figure 2. As it can be seen from the figure, samples 2 (NASHCl) and 4 (DND-30) have similar distribution as the biggest per cent of the particles (28%–30%) have average size of 56.25 and 51.10 nm, respectively. Samples 1 (NSFPA) and 3 (YTM) are larger in size with around 20% of particles with size of 103.20 and 233.82 nm, respectively. These results suggested aggregation of DND particles because, as mentioned above, the typical size of DND particles is between 2 and 10 nm.

Determination of the metal impurities in the DNDs was done using a direct method based upon the direct aspiration of aqueous ND suspensions into a sector field ICP-MS. This novel method was recently described by Mitev et al.[19] Some of the impurities in the different DND samples are presented in Table 1. In general, elemental analysis revealed that NSFPA particles were free from impurities and non-diamond carbon. NASHCl had the same elemental characteristics as NSFPA, with slightly higher content of non-diamond carbon. YTM showed higher levels of Cr while DND-30 contains more Ba and Pb.

**Table 1.** ICP-MS analysis of DND samples.

	1	2	3	4
Sample no.	NSFPA	NASHCl	YTM	DND-30
B11 (LR)	21.41	5.87	6.38	15.65
Sr88 (LR)	0.33	1.08	9.13	39.93
Nb93 (LR)	0.37	1.22	4.93	0.4
Mo95 (LR)	1.18	4.56	44.32	5.64
Sn118 (LR)	0.09	1.27	36.86	81.02
Sb121 (LR)	26.58	72.91	0.39	1.48
Ba137 (LR)	176.8	261.36	49.86	3324.31
W182 (LR)	0.25	1.4	4.04	1.82
Pb208 (LR)	97.62	84.79	11.33	1475.11
Bi209 (LR)	0.02	0.03	0.13	0.01
Na23 (MR)	31.6	50.28	243.51	89.93
Mg24 (MR)	2.48	12.84	264.01	1.8
Al27 (MR)	12.71	65.86	459.73	28.94
Si28 (MR)	47.37	148.64	1427.4	319.36
P31 (MR)	0.18	1.39	3.46	2.85
S32 (MR)	388.4	467.9	763.62	2263.66
Ca42 (MR)	0	45.3	3013.76	36.19
Ti47 (MR)	396.01	1217.02	1281.43	28.32
V51 (MR)	5.13	13.64	27.68	0.85
Cr52 (MR)	5.76	4.78	5107.28	9.51
Mn55 (MR)	0.19	1.65	49.34	17.56
Fe56 (MR)	37.16	109.56	328.51	358.6
Ni60 (MR)	9.32	30.71	3.36	21.09
Cu63 (MR)	0.59	14.11	74.88	1.75
Zn66 (MR)	0	161.31	61.88	4.41
K39 (HR)	11.36	18.64	207.49	9.69

### Cytotoxicity study

Data on cytotoxicity of detonation ND particles show that they cause limited or no toxicity at a cellular level.[8] Our previous study with silicon- and silver-modified DND particles also demonstrated a negligible toxic effect on osteoblast-like cells.[20] Nevertheless, cytotoxicity and biocompatibility of new detonation ND particles must be tested since it is possible to be affected by the purification method, which can alter size, charge or other surface properties of the particles. Moreover, cytotoxicity can vary, depending on the type of the cells used for the *in vitro* study.[21] Here, we have studied the cytotoxicity of acid-purified DND particles with two cell models: an osteosarcoma cell line (MG-63) and primary rMSCs. To assess cytotoxicity we have characterized the ability of mitochondria to reduce tetrazolium salts – a parameter widely used in toxicological studies. The mitochondrial function and by extension the viability and proliferation of cells were measured by the means of CCK assay after culturing of the cells in the presence of nanoparticles for 72 hours.

**Figure 3.**
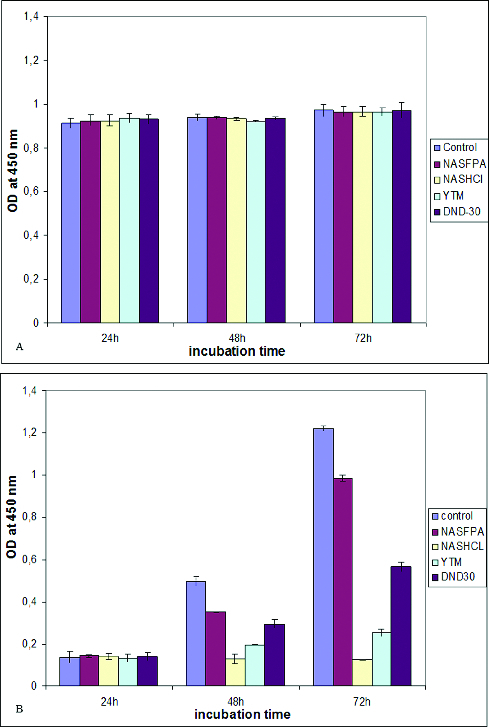
Proliferation activity of MG-63 cells (A) and rMSCs (B), incubated for three days with different DND particles.

The results indicated that, in general, both cell types have different sensitivity towards ND particles. As it can be seen from Figure 3, the cytotoxic effect of nanoparticles on mitochondrial activity was more evident in rMSCs, 48 hours after the exposure of the cells to nanoparticles. On the first day of incubation of the cells with different DND particles, the proliferation ability of both cell models, MG-63 and rMSCs, was not affected, even a slight increase in proliferation rate was observed compared to the control, but the differences were not statistically significant. However, with increasing of the incubation time an inhibition of mitochondrial function was observed in the presence of most of the particles. The strongest cytotoxic effect on mitochondrial activity of rMSCs had the NASHCl particles while in the presence of YTM particles rMSCs grew slightly and the most significant increase in cell proliferation was observed in the presence of NSFPA on the second and third days.

In MG-63 cells we observed a slight increase in growth rates on the thired day of incubation with DND particles. Keeping in mind that MG-63 cells are osteosarcoma cells characterized with high proliferation ability, it could be considered even as cytotoxic the effect of DNDs particles on MG-63 cells. On the other hand, the cancer origin of MG-63 cells made them more resilient to nanoparticles as it can be concluded from the slight difference between different types of nanoparticles. However, there were many exceptions, and a general statement relating cell activity and toxic response was not possible.

**Figure 4.**
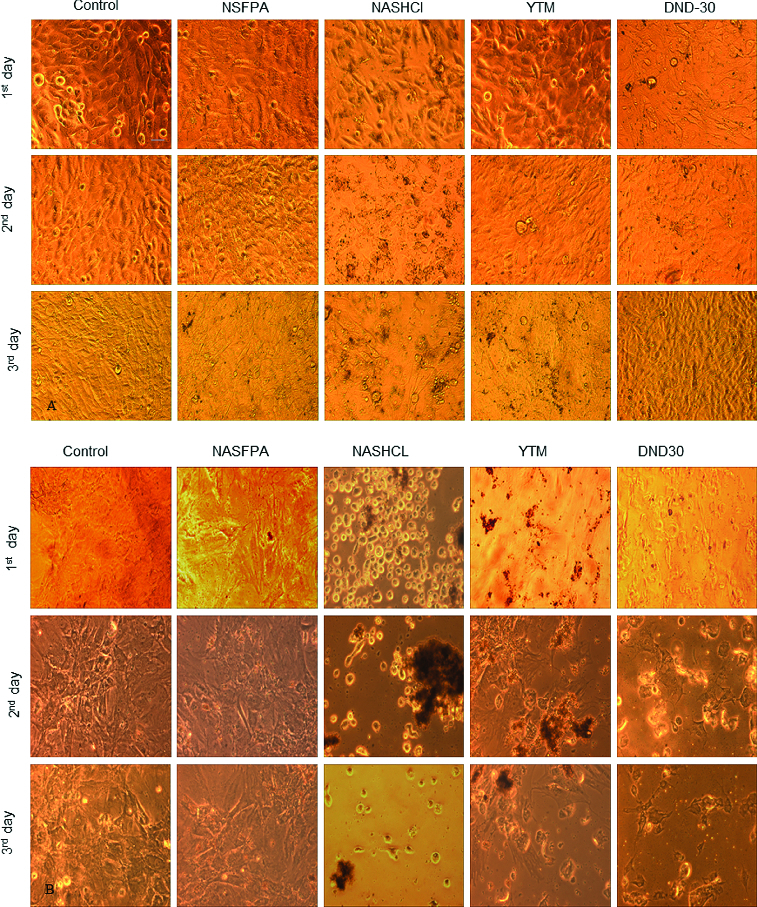
Overall morphology of MG-63 (A) and rMSCs (B) cells, incubated for three days with different DND particles, bar = 100 μm.

Overall morphology of rMSCs and MG-63 cells exposed to DND nanoparticles is shown in Figure 4. After incubation with different DND particles (0.1 g/ml) for 72 hours, MG-63 cells were well spread and their morphology was not noticeably different from the control cells. However, dramatic changes occurred with rMSCs. Within 24 hours of exposure to NASHCl particles, the cells started to round and detach from culture dishes while NSFPA did not induce any changes in cell morphology. In the presence of YTM and DND-30 particles cell detachment was observed after 48 hours of incubation with particles. This delayed toxicity remains unclear and needs further investigation. It is possible to have a correlation with the aggregation of DND particles, which increases with time of incubation. Phase-contrast imaging showed that DND particles tended to aggregate after the second day and the most aggregated particles showed the highest cytotoxicity. Agglomerates of NDs were visible in the surrounding media because the aggregates cannot translocate across the cell membrane due to their large size and stay outside of the cells. Interestingly, DND particles did not induce shrinkage, necrosis or apoptosis of the cells.

In summary, most toxic were the NASHCl particles containing non-diamond carbon because they suppressed cell proliferation and caused cell detachment on the 24th hours after their addition to the cells. The lowest cytotoxicity demonstrated NSFPA nanoparticles which had no impurities and YTM and DND-30 particles containing Cr and Ba and Pb, respectively, affected cell viability as well, although in a different degree.

## Conclusions

Our study confirmed that cytotoxicity of DND particles depended on complex particle properties, including size, purity and agglomeration. Furthermore, the cytotoxicity of detonation ND particles could be significantly affected by the cell type selected for the test. Therefore, care must be taken to choose the cells which are most relevant to the *in vivo* situation of interest, since the response of given cell line is likely to vary considerably.
